# Anatomically Veridical On‐Scalp Sensor Topographies

**DOI:** 10.1111/ejn.70060

**Published:** 2025-03-13

**Authors:** Nicholas A. Alexander, Johan Medrano, Robert A. Seymour, Stephanie Mellor, George C. O’Neill, Meaghan E. Spedden, Tim M. Tierney, Eleanor A. Maguire

**Affiliations:** ^1^ Functional Imaging Laboratory (FIL), Department of Imaging Neuroscience, UCL Queen Square Institute of Neurology University College London London UK; ^2^ Oxford Centre for Human Brain Activity (OHBA), Department of Psychiatry University of Oxford Oxford UK; ^3^ Spinal Cord Injury Center Balgrist University Hospital Zürich Switzerland; ^4^ Translational Neuromodeling Unit (TNU), Institute for Biomedical Engineering University of Zurich & ETH Zürich Zurich Switzerland; ^5^ Department of Neuroscience, Physiology and Pharmacology University College London London UK

**Keywords:** 10–20, data visualisation, EEG, magnetoencephalography, optically pumped magnetometer, topography

## Abstract

When working with sensor‐level data recorded using on‐scalp neuroimaging methods such as electroencephalography (EEG), it is common practice to use two‐dimensional (2D) representations of sensor positions to aid interpretation. Positioning of sensors relative to anatomy, as in the classic 10–20 system of EEG electrode placement, enables the use of 2D topographies that are familiar to many researchers and clinicians. However, when using another increasingly popular on‐scalp neuroimaging method, optically pumped magnetometer–based magnetoencephalography (OP‐MEG), bespoke sensor arrays are much more common, and these are not prepared according to any standard principle. Consequently, polar projection is often used to produce individual sensor topographies that are not directly related to anatomy and cannot be averaged across people simply. Given the current proliferation of OP‐MEG facilities globally, this issue will become an increasing hindrance when visualising OP‐MEG data, particularly for group studies. To address this problem, we adapted and extended the 10–20 system to build a flexible, anatomical projection method applied to digitised head shape, fiducials and sensor positions. We demonstrate that the method maintains spatially veridical representations across individuals improving on standard polar projections at varying OPM sensor array densities. By applying our projection method, the benefits of anatomically veridical 2D topographies can now be enjoyed when visualising data, such as those from OP‐MEG, regardless of variation in sensor placement as in sparse or focal arrays.

## Graphical abstract

Two‐dimensional sensor topographies are useful for inspecting data from on‐scalp sensors, such as optically pumped magnetometers used for magnetoencephalography. For bespoke arrays of sensors, polar projection methods can produce inconsistent results. Here we introduce an anatomical projection method which reproduces the 10–20 system digitally to ensure consistent, familiar topographies, regardless of array design or individual anatomy.

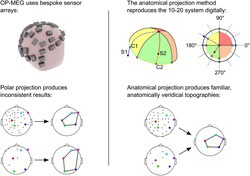

**Abbreviations**
2D: two‐dimensional
3D: three‐dimensional
EEG: electroencephalography
INI: inion
LPA: left preauricular
MEG: magnetoencephalography
MRI: magnetic resonance imaging
NAS: nasion
OPM: optically pumped magnetometer
OP‐MEG: OPM‐based MEG
ROI: region of interest
RPA: right preauricular
SPM: statistical parametric mapping

## 1 Introduction

Neuroimaging techniques such as electroencephalography (EEG) and magnetoencephalography (MEG) involve placing electrodes or sensors at fixed locations around the head to measure electromagnetic signals from the brain. When analysing these data, it is common to reduce the three‐dimensional (3D) sensor positions to two‐dimensional (2D) representations on a circle. These 2D representations can then be used to visualise the spatial topography of event‐related potentials, oscillatory power, or other data features and statistics. Regardless of subsequent analysis, which for MEG would typically be conducted in source space, these visualisation tools are essential during preprocessing and initial inspection of data (Gross et al. 2013). Additionally, sensor‐level data best preserves the high‐dimensionality required for multivariate analyses. As a result, a method for visualising sensor‐level outputs such as decoder weights or findings from spatial searchlight analyses is useful when interpreting results.

**FIGURE 1:**
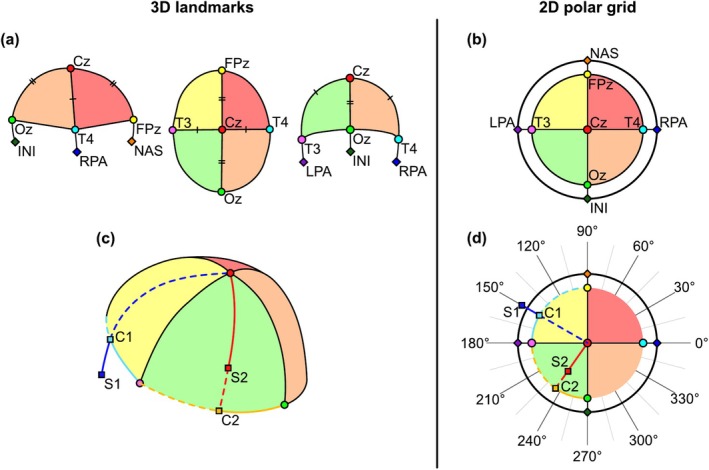
An overview of the 10–20 system transformation from 3D head shape to 2D polar grid. (a) Fiducials are marked and connected by lines crossing the scalp surface. Anatomical references (NAS, INI, LPA, RPA and Cz) are shown along with electrode placements that define the circumference of the polar grid shown in (b). Note, paired marks on lines indicate equidistance. (b) An illustration of the topography representing anatomical reference points. (c) Positions on the scalp (S1, S2) and their intersection with the circumference (C1, C2) are defined by a line from the vertex (Cz) to the scalp position, as marked. (d) This process is repeated on the 2D polar grid to represent scalp positions on the topography. NAS = nasion, INI = inion, LPA = left preauricular, RPA = right preauricular.

For interpretability, these 2D representations, or topographies, should be consistent and comparable across individuals, regardless of variation in head position, shape and size. For EEG, this is achieved by placing electrodes at specific, predefined locations, most commonly using the 10–20 system (Jasper 1958). This involves producing an axis across the scalp based on key anatomical landmarks around the head: the nasion (NAS), inion (INI), left preauricular (LPA) and right preauricular (RPA) points. Electrodes are then placed in locations relative to this axis and labelled accordingly. A 2D topography can be derived by applying the same electrode placement procedure onto a 2D axis overlaid onto a circle that represents the head (see Figure 1). This procedure has been extended with higher density variations, such as the 10–5 system (Oostenveld and Praamstra 2001) and, while other EEG electrode layouts exist (Seeck et al. 2017), the same 3D to 2D projection is typically employed. By using this approach, anatomically veridical 2D summaries of 3D electrode positions on the scalp can be produced that are consistent across individuals. This allows group‐level statistics, such as averaging, to be applied based on 2D topographical positions alone.

For fixed sensor arrays, such as those used for cryogenic MEG, sensor positions cannot be adjusted according to individual anatomy (Knösche 2002). To produce 2D representations of the 3D sensor positions, a polar projection is typically used. Usually this is adjusted to achieve a top–down view of the head, with a nose superimposed to face upwards and sensors evenly spaced around a 2D circle representing the head. Although some congruence is likely, it cannot be assumed that data from a specific sensor position directly relates to the anatomy it appears to represent. Despite consistent positioning of participants, varying head shape, size and position between datasets inevitably shift the location of neuromagnetic signals relative to sensors. Without correction to a common space (Ashburner and Friston 1997; Knösche 2002), or estimating a virtual sensor array (Knösche 2002), group‐level statistical analysis using 2D sensor‐level topographies produced using polar projection will be conservative—or less sensitive—due to unknown, nonlinear spatial smoothing across participants (Gross et al. 2013).

These issues need not necessarily apply to all fixed arrays. A new generation of wearable MEG sensors, optically pumped magnetometers (OPMs), have been developed that do not require cryogenic cooling (Boto et al. 2017) and this allows them to be placed much closer to the scalp in bespoke, on‐scalp arrays. However, these arrays are not typically placed according to prespecified montages such as the 10–20 system. The arrangement of OP‐MEG sensors can vary widely, from arrays where sensors are approximately equidistant from each other covering the whole head to sparse, targeted coverage designed to image a specific area of the brain (Hill et al. 2020; Tierney et al. 2018). One approach in OP‐MEG is to use bespoke 3D‐printed scannercasts to hold arrays of OPM sensors based on the structural MRI of an individual participant (Boto et al. 2018). A more recent alternative is to use generic helmets of fixed or semi‐fixed arrays (Alem et al. 2023; Brookes et al. 2022). OPMs combined with optical coregistration (Zetter, Iivanainen, and Parkkonen 2019) have also been used to scan larger cohorts (Rhodes et al. 2023). In all these cases, there is currently no method that accounts for individual anatomy when producing 2D topographies from 3D sensor positions, and therefore, OP‐MEG suffers from the same problems as fixed sensor arrays when using 2D representations.

Further, in those situations where OPM sensors do not cover the whole head, are placed in asymmetric positions or vary in number between recordings (Brookes et al. 2022; Hillebrand et al. 2023; Seymour et al. 2021), existing methods based on polar projections will produce inconsistent results, and sensor‐level group analysis will be suboptimal, as we will show. While it is possible to correct to a common space for OP‐MEG, this requires additional inverse modelling and in doing so directly alters the sensor‐level data rather than just their visualisation. Furthermore, non‐linear warping may not be suitable for sparse spatial samples. Given the current proliferation of OP‐MEG facilities globally, this issue will become an increasing hindrance to group studies.

Here we outline a solution to this problem by relating OPM sensor positions to known anatomical landmarks. To do this, we have adapted ideas from the widely used 10–20 system to develop a computationally efficient method of 3D to 2D transformation that requires no manual adjustment and works for any number of sensors placed anywhere on the head, regardless of sparsity. This results in anatomically veridical 2D topographies that are consistent across people, to the same standard achieved in EEG, without requiring a predetermined sensor placement protocol. In the following sections, we will describe this anatomical projection method, compare it to polar projection and demonstrate its practical use in OP‐MEG data analysis using simulations.

## 2 Materials and Methods

### 2.1 The 10–20 System

The methods presented here involve the reverse engineering of the widely used 10–20 system to first define a 3D grid on a digitised head shape and then apply that to a 2D polar grid. Below, we briefly describe the steps of the 10–20 protocol relevant to our method (see Figure 1). For a comprehensive overview of the 10–20 protocol, see Jasper (1958).

The 10–20 system of electrode placement requires only a pen and a tape measure to assess and mark specific positions on the head. Initially, four anatomical landmarks (fiducials) identified: NAS, INI, LPA, RPA. Subsequently, measurements are made across the scalp from NAS to INI and from LPA to RPA, and a central point at the vertex (Cz) is marked where Cz to NAS and Cz to INI are equidistant, as should be Cz to LPA and Cz to RPA (Figure 1a).

A complementary 2D polar grid is also formed with Cz at the origin and the circumference defined by each fiducial separated at 90° (Figure 1b). Positions on the scalp can then be described by their relation to these reference positions (Figure 1c,d). For example, Point S2 on Figure 1c can be located on the scalp as approximately located at 40% of the distance along the scalp between INI and LPA (Point C2) and approximately 60% of the distance along the scalp between Cz and C2. The corresponding 2D position of the 3D point is determined by applying this relative definition to the 2D polar grid (Figure 1d), that is, at 40% of the angle between INI and LPA and at 60% of the full radius. The underlying principle of our anatomical projection method is that any point on the scalp can be retroactively defined from its relation to anatomical points on the head.

### 2.2 Anatomical Projection Method

We have adapted and expanded the 10–20 system to produce an anatomical projection method. This involves first defining individual anatomy based on fiducial landmarks and then automatically measuring distances across the surface of the scalp to define the 10–20 polar grid. Next, the position of each OPM sensor is measured relative to this grid and those relative measures are reported to a 2D polar grid, following the same procedures as the 10–20 system.

**FIGURE 2:**
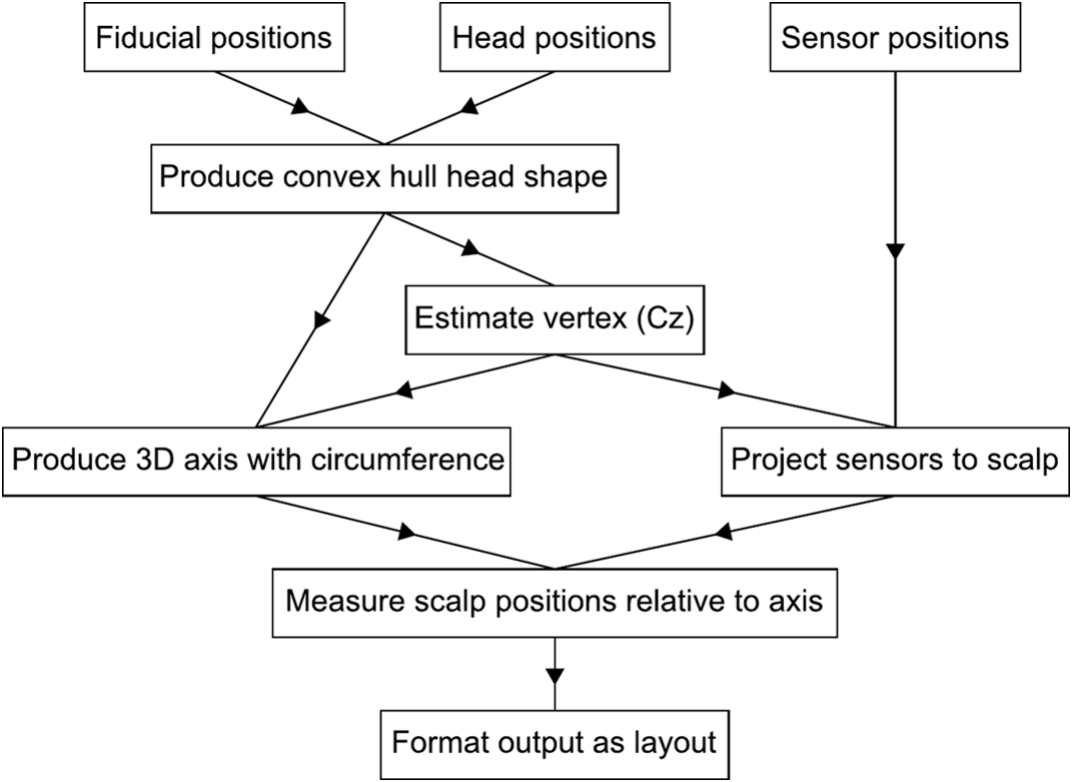
Flowchart showing a high‐level overview of the anatomical projection method. This protocol requires only the input of fiducial, head and sensor positions, with no further manual steps.

A summary flowchart of the steps involved in anatomical projection is shown in Figure 2, and full details are provided in the next sections. The method has been added to the Statistical Parametric Mapping (SPM, www.fil.ion.ucl.ac.uk/spm) MATLAB toolbox as a single function, *spm_get_anatomical_layout*, available in the SPM 25.01 release (Tierney et al. 2025).

#### 2.2.1 Requirements

Anatomical projection requires the user to provide sensor positions, head shape and fiducials, which must be registered in the same coordinate system. The precise method to acquire this information will vary between OP‐MEG researchers but may include spatial coregistration of structural MRI and a 3D optical scan of the participant wearing the sensor array (Zetter, Iivanainen, and Parkkonen 2019) or automatic coregistration of a 3D model of a sensor array and structural MRI (Meyer et al. 2017).

For spatial coregistration of sensor positions with the brain for bespoke setups, such as in OP‐MEG systems described above, information about NAS, INI, LPA and RPA positions are often required. If these landmarks have not already been measured directly, they are available from a 3D optical scan or structural MRI scan.

To use anatomical projection, the user must input this information as a set of points on the head and labelled fiducials. These points are then combined, and a 3D mesh is produced using a convex hull method. This produces a triangular mesh at the outer boundary of the points provided by preventing any concave edges. In doing so, irregularities or deviations in head shape are discarded, just as they would be if a tape measure was used to measure across the scalp. Furthermore, when the ears are included within the head positions—which is often the case when stripping the scalp from a structural MRI scan—errors in measurements from the vertex to below the ear are reduced.

#### 2.2.2 Measuring Along Head Surface

**FIGURE 3:**
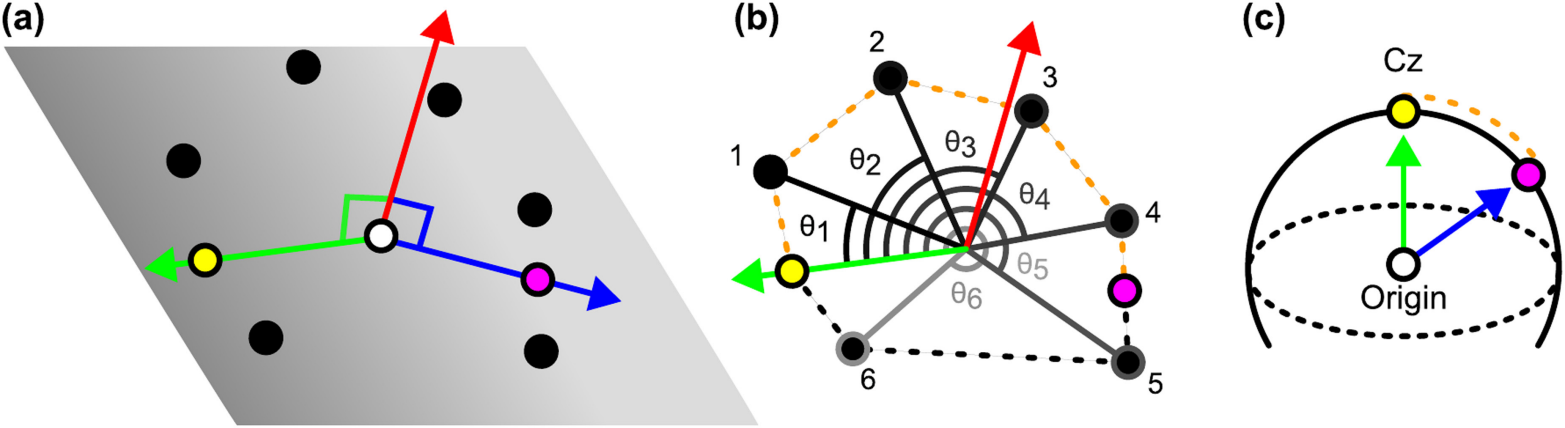
Visual explanation of how to measure across a convex mesh surface using a plane intersection method. (a) A plane is defined based on three points: two points on the mesh surface (yellow and magenta dots) and the origin (white dot). The normal of these three points (red arrow) defines a plane, shown by the grey gradient. Points where this plane intersects with the mesh are shown by the black dots. (b) In order to measure along the mesh from yellow to magenta, the positions must be indexed. This is achieved by measuring the polar angle (θ) to one of the points—in this case yellow—orthogonal to both the plane normal and a vector from the origin to each point. With the points indexed, a cumulative sum of distances between points from yellow to magenta (orange dashed line), following a left‐hand rule, can be taken. (c) An example of applying this method of measuring across the surface to find the distance from Cz to a position on the scalp. The dotted line represents the circumference of the head.

To digitally replicate the 10–20 system, it is necessary to accurately measure distances along the scalp akin to the pen and tape measure method. A visual explanation of the method used can be seen in Figure 3. Any two points on the scalp are crossed by a unique plane that also crosses the origin, where we define the origin as the midpoint between the positions LPA and RPA (i.e., the centre of the head). To measure the distance between two points on the scalp, we compute their origin‐crossing plane and extract the intersections of that plane with the 3D head mesh (Figure 3a). As the 3D mesh is convex, its intersection with any plane yields a 2D convex set of points (de Berg et al. 2008). This characteristic enables us to order the points along the intersection plane based on their polar angle around the plane normal vector at the origin. This order is strict; in other words, no two points have the same index. Having established this order, we can approximate the on‐scalp distance between Points A and B by summing up the Euclidean distances between all pairs of consecutive points whose indices are between that of A and B (Figure 3b). Importantly, the accuracy of this piecewise approximation increases with the mesh resolution, but even a relatively low‐resolution mesh would be sufficient in this case. In the following sections, we show how this method was systematically used to measure the distance between points on the head (Figure 3c).

#### 2.2.3 Definition of 10–20 Polar Grid

To define the 10–20 polar grid, we start by constructing the vertex point of the scalp, Cz. A first estimate of its location is obtained by finding the intersection through the scalp mesh of a line crossing through the origin and directed along a vector orthogonal to vectors taken between each of the fiducial points. This estimation is then refined. To do so, we first compare measurements along the scalp from the estimated Cz position to each pair of fiducials (LPA and RPA; NAS and INI) to check whether they are equidistant, within‐pair. If, for example, the distance from Cz to LPA is greater than that from Cz to RPA, an adjustment is made. This is done by translating the vector estimating Cz towards the fiducial with the greater distance, in this case LPA. This process is repeated until equidistance is achieved, to a tolerance of 1 mm. After convergence, the vertex point Cz is added to the mesh.

Once fiducial points have been measured, the next step is to shorten the measurement across the scalp from vertex to fiducial by 20%, giving the points T4, FPz, T3 and Oz. This follows standard application of the 10–20 system. Next, the circumference of the head is marked by connecting these new endpoints. This, in essence, follows the same procedure described in the 10–20 system section above and shown in Figure 1a.

#### 2.2.4 Redefining Sensor Positions

With a model of the head and landmarks defined according to the 10–20 system, the next step is to redefine each 3D OPM sensor position within this system. Figure 1c shows this process whereby each sensor must be described by its relative distance along the scalp from the vertex to the circumference and its alignment along the circumference.

To achieve this, each OPM sensor position is projected onto the scalp surface using a ray cast method from the sensor position to the origin (see Section 4.1 for an evaluation of this approach). Subsequently, we measure the on‐scalp distance from Cz to the sensor. This measure is done by applying the procedure described in Section 2.2.2, in particular, by computing a *measurement plane*, crossing the sensor location on the scalp, the vertex Cz and the origin. We then measure along the same measurement plane the distance from Cz to the circumference. The ratio of these two distances determines its relative position between Cz and the head circumference; in other words, how eccentric the 2D sensor position will be.

The next step is to determine the polar angle. Here we depart substantially from polar projection by determining the polar angle as a ratio of distance between points. To do this, we first find the point on the circumference crossed by the measurement plane. A measurement is then made along the circumference from the point the circumference was crossed to the nearest fiducial in either direction; for example, T3 and Oz, or FPz and T4. The ratio of these two distances is used to determine the polar angle, with an initial offset determined by the fiducial pair. These two measures are used to generate a 2D position following the principles of the 10–20 system.

Note that sensors may be placed in positions more eccentric to the vertex than the defined circumference. For example, OP‐MEG arrays may cover the face or even the mouth (Mellor et al. 2023; Tierney et al. 2021). In such cases, the same measurements are taken and, where the ratio is greater than 1, the position will be defined outside the circle. An example of this process can be seen in Figure 1c,d. In the case of Sensor Position 1 (S1), the on‐scalp distance from Cz to S1 is 18 cm. The on‐scalp distance from Cz to C1 is 12 cm. In this case, S1 would be placed at a distance of 18/12=1.5 units away from the pole. As before, polar angle is calculated by first measuring the on‐scalp distance from FPz to C1 (10 cm) and FPz to T3 (15 cm) and then taking their ratio ($5/15=0.\dot{6}$). This ratio is then applied to an initial offset from FPz ($90^{{}^{\circ}}+\left(90^{{}^{\circ}}*0.\dot{6}\right)=150^{{}^{\circ}}$). This approach aligns with other expansions of the 10–20 system, such as the 10–5 (Oostenveld and Praamstra 2001), which extend further away from Cz.

### 2.3 Testing Anatomical Projection

To test anatomical projection, we used 24 sets of head geometry, fiducials, sensor positions and structural MRI scans obtained from an ongoing OP‐MEG study. Each participant provided written informed consent, and the study was approved by the University College London Research Ethics Committee (Project ID 6743/007). No original OP‐MEG data were used; all data presented in this article were generated based on the geometry described.

From these 24 datasets, we produced 2D layouts using anatomical and polar projection under varying conditions, described below. Layouts based on polar projection were calculated using the function *ft_prepare_layout*, implemented in Fieldtrip (Oostenveld et al., 2010) with parameters set to centre and rotate the array to match the typical ‘nose up’ format of the 10–20 system. In our case, parameters were consistent for all 24 datasets, as they had previously been aligned to the same orientation. Anatomical projection topographies were calculated using our new function *spm_get_anatomical_layout*, as implemented in SPM.

To assess the performance of anatomical versus polar projection, we estimated leadfields in common MNI space for each subject's head geometry. A cortical atlas, also in MNI space (Desikan et al. 2006), was then used to define voxels for 56 regions of interest (ROIs) in each subject. For each ROI, we calculated the mean magnitude of the leadfields within the current ROI, resulting in one value per channel. These data were then applied to layouts generated under different conditions.

## 3 Results

### 3.1 Comparison of Anatomical and Polar Projection

One of the key features of anatomical projection is that the 2D representation of each sensor is computed independently of other sensors. Therefore, regardless of the number or position of other sensors on the head, the 2D position of a given sensor will be the same. This is not the case for standard polar projection methods which build up a polar axis from the whole array.

We demonstrate the benefits of our approach by comparing its performance with polar projection using the positions of 40, 20, 10 and 5 sensors distributed across a scalp surface. Sensor positions, head shape and fiducials have been taken from a single OP‐MEG whole‐head fixed‐array dataset.

**FIGURE 4:**
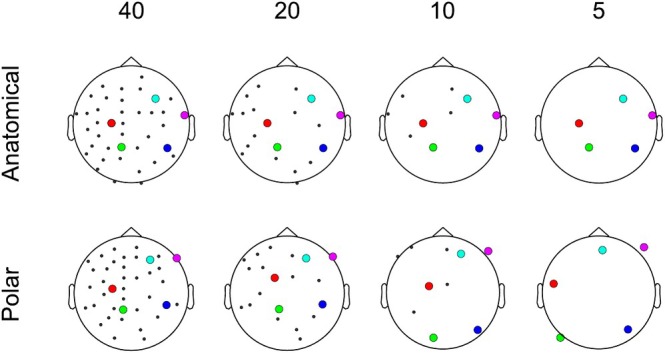
Comparison of anatomical and polar projection using varying numbers of OPM sensors on the head. The top panel (anatomical projection) shows that highlighted sensors are consistent across all conditions. However, in the lower panel (polar projection), highlighted sensor positions vary in each sensor count condition, producing unreliable results.

Figure 4 shows the results of this comparison. As expected, OPM 2D sensor positions calculated using anatomical projection remain stable regardless of the number of sensors. For 40 sensors with approximately whole‐head coverage, polar projection performs adequately (after manual adjustment and without consistency between sensor number conditions, discussed earlier). However, as the OPM sensor number decreases, sensor positions diverge. This is undesirable, because spatial interpretation of signals based on 2D topographies produced using polar projection will not reflect their true position. For example, the sensor labelled in green should be located at a left central–posterior position, but as the sensor count decreases, it moves to an eccentric, posterior position, which could result in misinterpretation of visualised data.

### 3.2 Anatomical Consistency Improves Group Statistics at Sensor Level

Another benefit of anatomical projection is that it allows for the averaging of output across datasets containing different numbers of sensors in various positions, without altering the original data. While this process can also be applied to polar projections, it is not recommended for sparse or focal arrays, as we will demonstrate.

**FIGURE 5:**
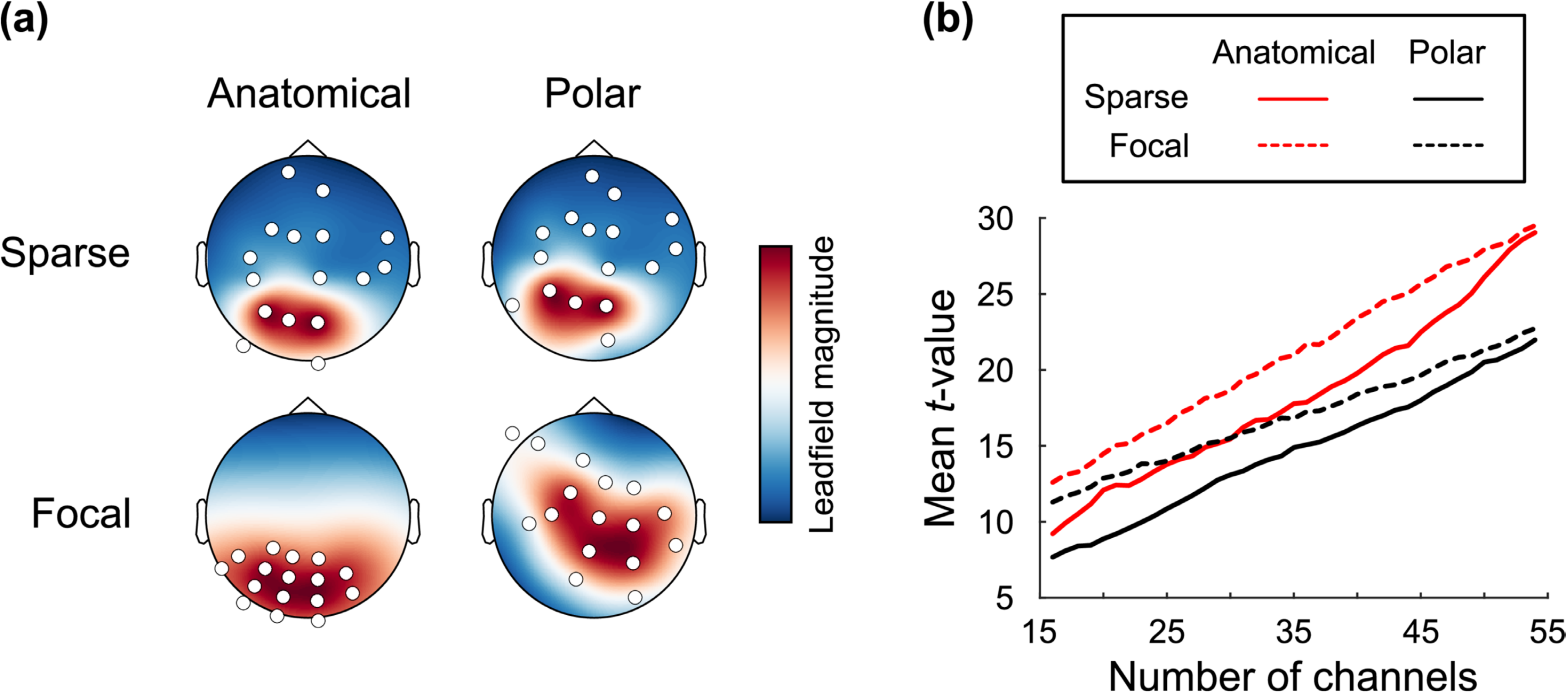
Performance of anatomical projection versus polar projection with varying number of channels under sparse and focal conditions. (a) An example of the data generated for this simulation based on the left cuneus ROI (LH_cuneus). Data based on a single subject's head geometry and sensor positions are shown having been used to produce a layout for 16 channels either randomly selected (sparse) or selected based on proximity to the channel with maximum leadfield magnitude (focal). (b) A summary of the mean *t‐*value (across ROIs and topographical grid) by number of channels under the conditions shown. Anatomical projection consistently outperformed polar projection, regardless of sparsity/focality and number of channels.

Anatomical and polar projection layouts were created based on subselections of real sensors. Initially, we selected sensors covering the whole head (54 channels), created an anatomical and polar projection layout based on these 54 channel positions and then generated a topographical image based on the leadfield magnitudes, resulting in (number of subject) by (number of ROI) 2D topographical images. These images were normalised using a z‐score within‐subject based on all values within the interpolated grid of leadfield magnitudes. Next, for each ROI, *t*‐values across subjects were calculated for each grid position. This procedure is visualised and demonstrated in Figure 5a.

We repeated this process across two pipelines. First, to simulate how array sparsity affects anatomical versus polar projection methods, we decreased the number of channels in steps of one from 56 down to 16 channels, by randomly removing one channel position at a time. Second, to simulate how varying array focality affects anatomical versus polar projection, we again decreased the number of sensors from 56 to 16 in steps of one but did not remove them randomly. Instead, we identified the channel with the peak leadfield magnitude for the current ROI and then selected the nearest *N−*1 channels, where *N* was the desired number of channels to keep.

The results of these analyses are presented in Figure 5b. For both sparse and focal sensor layouts, the mean *t*‐value for group averages created using anatomical projection was consistently higher than that created using polar projection, regardless of the number of channels. Supporting the demonstration shown in Figure 4, sparse and focal arrays were found to be unrepresentative of 3D sensor position when polar projection was used but remained consistent with anatomical projection, as the position of channels in 2D did not depend on the rest of the array.

## 4 Discussion

Here we have introduced a method, anatomical projection, for obtaining 2D representations of 3D OP‐MEG channel positions based on individual anatomy. By generalising and extending the protocols applied in the classic 10–20 system of EEG electrode placement, we have enabled automatic representation of OPM sensor positions. Consequently, the benefits of anatomically valid 2D topographies, widely used in analysis and dissemination of EEG findings, can now be applied to OP‐MEG and other on‐scalp neuroimaging techniques.

Currently, the most pressing use case for the anatomical projection method is in OP‐MEG, where constant advancements in sensor systems, now incorporating as many as 174 channels (Rhodes et al. 2023), have led to an increase in data collection capacity. OP‐MEG publications often present sensor‐level data for individual participants separately (Seymour et al. 2021) in part due to the challenge of averaging across bespoke OPM sensor positions. Note, the use of anatomical projection is not limited to on‐scalp, bespoke sensor arrays. It could also improve sensor‐level presentation of generic, helmet‐based OP‐MEG or rigid sensor arrays placed close to the scalp, as in cryogenic MEG. This overcomes existing limitations with polar projections (see Figure 4) or similar methods that do not account for individual anatomy.

While MEG enjoys relatively high spatial resolution, enabling reliable analysis in source space, the use of sensor‐level representations remains common in preprocessing pipelines for various purposes. For example, when identifying bad channels or interpreting the results of spatial filtering techniques like independent component analysis across subjects (Cotroneo et al. 2024). Further, multivariate analyses are likely to be conducted at sensor level. To interpret spatial components of these analyses, such as decoder weights across cryogenic‐ or OP‐MEG channels (Bezsudnova, Quinn, and Jensen 2024; Zubarev et al. 2019), a visualisation tool is required. Anatomical consistency aids such uses and also the interpretability of results in the context of other research findings and across individuals. For instance, when evaluating epilepsy, sensor‐level topographies may be used to identify features for seizure localisation (Feys et al. 2022). Therefore, our proposed method offers significant benefits to MEG research by providing reliable and anatomically consistent 2D topographies.

Our method has other potential applications beyond OP‐MEG or cryogenic MEG, based on its ability to digitally recreate the 10–20 system of EEG electrode placement. For instance, it could be employed to aid automatic labelling of EEG electrode digitisation based on head shape and fiducials. Alternatively, it could be used to produce virtual MEG channels (Knösche 2002; Marhl et al. 2022) representative of familiar electrode positions to improve the interpretability of results to those more familiar with EEG. This is particularly beneficial when working with data from concurrent multimodal recordings, such as EEG and OP‐MEG (Seedat et al. 2024), or when comparing data from the same individuals recorded using different neuroimaging systems (Gutteling et al. 2023; Rhodes et al. 2023). Moreover, this method could facilitate the assessment of deviations between actual and ideal electrode placement, which commonly occur when using EEG caps designed to fit a broad range of individuals (Atcherson et al. 2007). By providing a better measure of this deviation, corrections can be made before conducting group‐level analysis at sensor level. Furthermore, anatomical projection allows the representation of bespoke EEG electrode placements, where whole‐head coverage may not be necessary or feasible and where alternative methods, such as polar projection, are not recommended.

### 4.1 Limitations

There are several points to consider when applying anatomical projection. The main limitation is the requirement for sensor position, head shape and fiducial information which, while normally available for MEG, may require additional steps to calculate. There are several ways to mitigate this. First, where sensor positions cover approximately the whole head and are near to the scalp, their positions can be treated as head positions, if moved inwards by the approximate distance from scalp to sensor (Homölle and Oostenveld 2019). Second, where only the NAS, LPA and RPA are measurable, but the INI is not, for example, due to occlusion when taking a 3D optical scan, the SPM implementation of anatomical projection can estimate the INI position from the remaining fiducials.

Another limitation of anatomical projection is that, while it accepts sensor position inputs of all kinds, the results will be most consistent when sensor arrays are near to the scalp and where distances from scalp to sensor are consistent across the whole array. Therefore, for fixed array systems, such as cryogenic MEG and some generic helmet‐based OP‐MEG (Rhodes et al. 2023), improvements from anatomical projection may be limited.

Finally, topographies produced by anatomical projection (and other existing methods) take no account of OPM sensor orientation. Generally, topographical plots of MEG data are made using channels at orientations axial to the head. However, dual‐ and tri‐axial OPM sensors such as QuSpin Gen‐2 and Gen‐3 zero‐field magnetometers (QuSpin, Colorado, United States) can also measure fields tangential to the head. Further developments in data visualisation methods are required which account for these additional channels such that the information recorded by them can be inspected and reported with the benefits offered by simple 2D representations.

### 4.2 Conclusion

In this technical note, we introduce anatomical projection—a method to generate participant‐specific 2D topographies of OPM sensor positions, drawing inspiration from the well‐established 10–20 system used in EEG. The main benefit, compared with existing methods, is that anatomical projection produces 2D topographies that are both consistent across people and datasets, as well as being anatomically faithful to the underlying neurophysiology. Our method is versatile and easy to use, requiring only four anatomical landmarks in addition to the OPM sensor positions and head shape. It can be used for various on‐scalp neuroimaging techniques but will be particularly useful for group‐level sensor‐space OP‐MEG data.

## Author Contributions

**Nicholas A. Alexander:** conceptualization, funding acquisition, software, visualization, writing – original draft, writing – review and editing. **Johan Medrano:** conceptualization, software, visualization, writing – original draft, writing – review and editing. **Robert A. Seymour:** conceptualization, software, visualization, writing – original draft, writing – review and editing. **Stephanie Mellor:** software, writing – review and editing. **George C. O’Neill:** software, writing – review and editing. **Meaghan E. Spedden:** writing – review and editing. **Tim M. Tierney:** software, writing – review and editing. **Eleanor A. Maguire:** funding acquisition, supervision, writing – original draft, writing – review and editing.

## Conflicts of Interest

The authors declare no conflicts of interest.

### Peer Review

The peer review history for this article is available at https://www.webofscience.com/api/gateway/wos/peer‐review/10.1111/ejn.70060.

## Data Availability

The anatomical projection method has been added to the SPM MATLAB toolbox as a single function *spm_get_anatomical_layout*, available in the SPM 25.01 release (Tierney et al. 2025). See https://github.com/spm/spm for the latest version, documentation and examples.
